# Histone Deacetylase Inhibitors as Anticancer Drugs

**DOI:** 10.3390/ijms18071414

**Published:** 2017-07-01

**Authors:** Tomas Eckschlager, Johana Plch, Marie Stiborova, Jan Hrabeta

**Affiliations:** 1 Department of Pediatric Hematology and Oncology, 2nd Faculty of Medicine, Charles University and University Hospital Motol, V Uvalu 84/1, Prague 5 CZ-150 06, Czech Republic; plchova.johana@gmail.com (J.P.); janhrabeta@gmail.com (J.H.); 2 Department of Biochemistry, Faculty of Science, Charles University, Albertov 2030/8, Prague 2 CZ-128 43, Czech Republic; stiborov@natur.cuni.cz

**Keywords:** histone deacetylases, histone deacetylase inhibitors, cancer, apoptosis, autophagy, cell cycle arrest, anti-angiogenic effect, drug combinations

## Abstract

Carcinogenesis cannot be explained only by genetic alterations, but also involves epigenetic processes. Modification of histones by acetylation plays a key role in epigenetic regulation of gene expression and is controlled by the balance between histone deacetylases (HDAC) and histone acetyltransferases (HAT). HDAC inhibitors induce cancer cell cycle arrest, differentiation and cell death, reduce angiogenesis and modulate immune response. Mechanisms of anticancer effects of HDAC inhibitors are not uniform; they may be different and depend on the cancer type, HDAC inhibitors, doses, etc. HDAC inhibitors seem to be promising anti-cancer drugs particularly in the combination with other anti-cancer drugs and/or radiotherapy. HDAC inhibitors vorinostat, romidepsin and belinostat have been approved for some T-cell lymphoma and panobinostat for multiple myeloma. Other HDAC inhibitors are in clinical trials for the treatment of hematological and solid malignancies. The results of such studies are promising but further larger studies are needed. Because of the reversibility of epigenetic changes during cancer development, the potency of epigenetic therapies seems to be of great importance. Here, we summarize the data on different classes of HDAC inhibitors, mechanisms of their actions and discuss novel results of preclinical and clinical studies, including the combination with other therapeutic modalities.

## 1. Introduction

Cancer chemotherapy has been one of the major medical advances in the last few decades. However, the drugs used for this therapy have a narrow therapeutic index, and the produced responses are often only palliative as well as unpredictable. Such approaches, although directed toward certain biomacromolecules, do not discriminate between rapidly dividing non-malignant and cancer cells. In contrast, targeted therapy that has been introduced in recent years is directed against cancer-specific targets and signaling pathways, and thus has more limited nonspecific mechanisms.

A crucial role for epigenetic mechanisms in cancer development is demonstrated by a number of studies. Carcinogenesis cannot be explained only by genetic alterations, but also involve epigenetic processes (DNA methylation, histone modifications and non-coding RNA deregulation). Histone modifications include H3 and H4 histones lysine deacetylation that leads to chromatin decondensation [1]. These alterations influence gene transcription including upregulation of several anti-oncogenes and DNA repair genes [2]. Thus, the epigenetic processes have emerged as novel therapeutic targets in numerous investigations.

The importance of histone deacetylase (HDAC) enzymes in organisms has been demonstrated using the studies with mice knocked out on members of class I HDACs. HDAC1-null mice die prenatally with severe proliferation defects and general growth retardation; HDAC2-null mice die the first day after birth for cardiac malformations; and HDAC3-null mice die prenatally for defects in gastrulation [3,4]. HDACs seem to be important for gene expression [5]. It has been described several times that their levels vary greatly in cancer cells and differ according to the tumor type. HDAC1 is highly expressed in prostate, gastric, lung, esophageal, colon and breast cancers [6,7,8]. High levels of HDAC2 were found in colorectal, cervical and gastric cancers [9,10]. In addition, HDAC3 is overexpressed in colon and breast tumors [11], whereas HDAC6 is highly expressed in mammary tumors, HDAC8 is overexpressed in neuroblastoma cells and HDAC11 mainly in rhabdomyosarcoma [12,13,14]. Increased expression of different HDAC and/or histone hyperacetylation in different cancers is caused by different mechanisms which may affect effects of individual HDAC inhibitors.

The in vitro analysis of different HDACs isoforms using siRNA (small interfering RNA) against HDAC1, HDAC2 and HDAC3 on several ovarian carcinoma cells (SKOV3, OVCAR3, IGROV-1, ES-2, TOV112D, A2780 and A2780/CDDP) showed that knockdown of HDAC1 inhibits proliferation and tumorigenicity, while knockdown of HDAC3 reduces cell migration with an increase in E-cadherin. On the contrary, knockdown of HDAC2 has no effect on proliferation and tumorigenicity, or cell migration [15].

In this review, we describe different classes of HDAC inhibitors and mechanisms of their actions, and discuss novel data found in several preclinical experiments and clinical studies. Moreover, we discuss their combination with other therapeutic modalities, particularly with DNA-damaging compounds.

## 2. Acetylation and Deacetylation

Modification of histones by acetylation plays a key role in epigenetic regulation of gene expression by changing the structure of chromatin and by modulating the accessibility of transcription factors to their target DNA sequences [16]. Histone acetylation is enriched in transcriptionally active regions of the genome, especially at proximal promoters and enhancers, and facilitates the binding of transcription factors. Acetylation loosens contact between core nucleosome proteins and DNA, thereby making transcription factor binding sites more accessible, recruitment of bromodomain proteins which recognize acetylated lysine residues on the N-terminal tails of histones and are actively recruited to enhancer and promoter regions [17]. The acetylation state of histones and other proteins is maintained by histone acetyltransferases (HAT) and HDAC. HATs catalyze the transfer of an acetyl group from acetyl-CoA to the ε-NH_2_ group of lysine residues in proteins, while HDACs remove it [12]. Acetylation status of non-histone proteins modifies many cellular functions e.g., mRNA splicing, transport and integrity; translation; activity, localization, stability and protein interactions [18]. Of the proteins whose functions are modulated by HDACs, the p53 (tumor protein 53), RUNX3 (Core binding factor α3 subunit), STAT3 (Signal transduction and activation of transcription 3), β-catenin, estrogen receptor, Myc (Avian myelocytomatosis viral oncogene homolog), EKLF (Erythroid Kruppel-like factor), GATA family (GATA-binding factors), HIF-1α (Hypoxia-inducible factor 1α), MyoD (Myogenic regulatory factor), NF-κB (Nuclear factor κB) or Foxp3 (Forkhead box P3 protein), are the most important in cancer (for review, see [19]).

Based on the homology to their yeast analogs, HDACs are divided into four classes. Class I, located in the nucleus, includes HDACs 1, 2, 3 and 8. HDACs 4, 5, 7 and 9 are members of class IIa, and class IIb (isoforms 6 and 10) are located in both cytoplasm and nucleus. Class IV (only the isoform HDAC11) exhibits some features of classes I and II. NAD^+^-dependent homologs 1–7 of the yeast Sir2 proteins (sirtuins) are designed as the class III of HDACs [20]. To remove the acetyl groups of proteins, the HDACs utilize two different mechanisms. These mechanisms act as the base for their classification into two distinct families. The “classical family” comprises of Zn^2+^-dependent HDACs (classes I, II and IV). The Zn^2+^ ion stabilizes the acetylated substrate in the catalytic center of the enzyme and polarizes the carbonyl group making the carbon to be a better target for nucleophilic water molecules [21]. The further HDAC family is NAD^+^-dependent, being capable of forming *O*-acetyl ADP ribose and nicotinamide as a result of the acetyl transfer [22].

Most of the classic HDACs form large complexes with multiple transcriptional co-repressors (such as Sin3 (Transcriptional regulatory protein SIN3), NuRD (Nucleosome remodeling and deacetylase complex), NcoR (Nuclear receptor co-repressor) and SMRT (Silencing mediator of retinoic acid and thyroid hormone receptors) with chromatin remodeling activity [23]. HDAC complexes can contribute to gene repression through two mechanisms—specific targeting by repressors and the constitutive association with chromatin [24,25].

HATs, which have the counterbalancing effect of HDAC, are a diverse set of multi-subunit complexes. They are divided into Gcn5 *N*-acetyltransferases (GNATs) and MYST HATs. GNATs include Gcn5 (General control non-repressed protein 5), PCAF (p300-CREB-binding protein-associated factor), Elp3 (Elongator protein 3), Hat1 (Histone acetyltransferase 1), Hpa2 (Hrp-associated 2 protein) and Nut1 (Component of the RNA polymerase II mediator complex). MYST is an acronym for the members of a protein family: Morf, Ybf2 (Sas3), Sas2 and Tip60. Although these two groups of HATs are the main ones, other proteins such as p300/CBP (CREB-binding protein), Taf1 (TATA box-binding protein associated factor 1) and a number of nuclear receptor coactivators show intrinsic acetylase activity. However, they do not contain true consensus HAT domains and therefore represent an “orphan class” of HAT enzymes [26].

## 3. HDAC Inhibition and Its Effects

Histone acetylation has been shown to be an important regulatory mechanism that controls transcription of approximately 2–10% of genes [27]. Deacetylation of histones causes chromatin condensation, while decondensation is caused by increased acetylation [1]. Such changes might result in decreased or increased gene transcription. However, there are other proteins than histones (e.g., chromatin remodeling proteins, DNA-binding nuclear receptors, DNA repair enzymes, signaling mediators, structural proteins, transcription coregulators, DNA-binding transcription factors) whose activity is affected by acetylation. In particular, the function of transcription factors can be positively or negatively affected. It provides an explanation why gene expression determined by HDAC inhibition, is not always increased even if the chromatin structure is loosened. The changes in HAT/HDAC activity balance can: (I) lead to an altered gene expression profile as well as to the change of some signaling pathways e.g., ERK (Extracellular signal-regulated kinase) and Wnt (Wingless/Int-1) pathways; (II) affect proteasomal degradation; (III) influence protein kinase C activity and (IV) change DNA methylation status. In various cancer cells, the shift to an increased acetylation/deacetylation ratio by HDAC inhibition was found to have a substantial effect on their fate (for overview see [19]).

HDAC inhibitors may act specifically against the only several types of HDACs (HDAC isoform-selective inhibitors), but also against all types of HDACs (pan-inhibitors). HDAC inhibitors can be classified as members of five classes of compounds: (I) hydroxamic acids (hydroxamates); (II) short chain fatty (aliphatic) acids; (III) benzamides; (IV) cyclic tetrapeptides; and (V) sirtuin inhibitors including the pan-inhibitor nicotinamide and the specific SIRT1 and SIRT2 inhibitors sirtinol and cambinol, respectively (for overview, see [28] and Table 1).

(I) Whereas trichostatin A (TSA) is an HDAC inhibitor used only in laboratory experiments because of its toxicity, vorinostat (suberoylanilide hydroxamic acid, SAHA) which has been already approved by United States Food and Drug Administration (FDA) as the first HDAC inhibitor, is utilized for the treatment of relapsed and refractory cutaneous T-cell lymphoma (CTCL). Belinostat (PXD-101) (approved for therapy of peripheral T cell lymphoma (PTCL)), panobinostat (LBH589) (approved for therapy of multiple myeloma) and the compounds recently tested in clinical studies, i.e., givinostat (ITF2357), resminostat (4SC201), abexinostat (PCI24781) and quisinostat (JNJ-26481585), are the pan-HDAC inhibitors. Selective HDAC inhibitors in the group of hydroxamic acids, including rocilinostat (ACY1215), which is selective inhibitor of HDAC class II, as well as practinostat (SB939) that inhibits all classical classes of HDACs (I, II and IV) and CHR-3996, the selective inhibitor of class I, are under clinical studies.

(II) The short chain fatty acids, valproic acid (VPA), butyric acid and phenylbutyric acid, are known to be weak inhibitors of HDAC class I and IIa and I and II, respectively. VPA is registered for the therapy of epilepsy, bipolar disorders and migraines, and is now together with other short chain fatty acids HDAC inhibitors tested in clinical studies as anticancer drugs.

(III) Among the benzamides that are now tested in clinical studies, entinostat (MS-275-SNDX-275), tacedinaline (CI994) and 4SC202 inhibit the class I HDACs. Mocetinostat (MGCD0103) is a selective inhibitor of classes I and IV HDACs.

(IV) The cyclic tetrapeptides include the bicyclic depsipeptide romidepsin (FK228, FR901228), a compound that has been approved by FDA and EMA (European Medicines Agency) to treat CTCL. It is a prodrug, which is reductively activated to a metabolite containing a thiol group that chelates the zinc ions in the active center of the HDAC of class I [29].

(V) Sirtuin inhibitors include the pan-inhibitor nicotinamide and the specific SIRT1 and SIRT2 inhibitors sirtinol, cambinol and EX-527. They might act against different types of neurodegenerations and cancers [30].

The other different HDAC inhibitors and the mechanisms of their actions are reviewed elsewhere (see [31,32,33,34,35,36,37]). Many various compounds with HDAC inhibiting activity are now being preclinically investigated.

## 4. Mechanisms of HDAC Inhibitors Action

HDAC inhibitors induce cancer cell cycle arrest, differentiation and cell death. Moreover, they reduce angiogenesis and modulate immune response. Hypothesis of “epigenetic vulnerability of cancer cells”, which has been proposed by Dawson and Kouzarides [38], is a cause of relative specificity of HDAC inhibitors. This hypothesis supposed that normal cells have in contrast to some cancer cells multiplied epigenetic regulatory mechanisms. Therefore, HDACs may be essential for the maintenance of a set of key genes required for survival and growth of cancer cells but not of normal ones [38].

Mechanisms of anticancer effects of HDAC inhibitors are not uniform; they may be different and depend on a type of cancer, on the individual HDAC inhibitor and its dose as well as on some other factors [39]. For example, VPA has shown to inhibit the invasiveness in bladder cancer but not in prostate cancer cells [40], while it has not induced cell cycle inhibition in some neuroblastoma cell lines such as SH-SY5Y and SK-N-BE [41].

### 4.1. Cell Cycle Arrest

Cell cycle arrest induced by HDAC inhibitors is caused by several mechanisms; the most important seems to be the increased expression of cell cycle genes such as *CDKN1A* (Cyclin dependent kinase inhibitor *p21*) as described in a variety of cancer cells [42,43,44]. Its product blocks the formation of dimers from cyclins and cyclin dependent kinases (CDKs) that induce cell cycle arrest and inhibition of cell differentiation [43,44]. The expression of p21 is modulated by protein p53 that interacts with *p21* promoter, competing with HDAC1, which decreases transcription of *p21* [45]. After treatment with HDAC inhibitors, the HDAC1 protein is released from the Sp1 (Promoter-specific RNA polymerase II transcription factor), which increase *p21* expression. Furthermore, HDAC inhibition increases acetylation of the p53 protein which results in an increase in its half-life [46], thereby improving the interaction with the *p21* promoter [47]. Moreover, the p53 protein interactions with its activators ASPPs (Ankyrin-repeat-, SH3-domain- and proline-rich region containing proteins), 53BP1 (p53-binding protein), TiP60/hMOF (Human males absent on the first), hCAS/CSE1L (Cellular apoptosis susceptibility protein), and HZF (Hematopoietic zinc finger) are regulated by its acetylation status which is influenced by HDAC inhibitors [48]. Finally, the p21 levels are increased, thereby mediating cell cycle arrest and apoptosis [43,49,50]. HDAC inhibitors can also inhibit expression of genes coding cyclin D and cyclin A resulting in the absence of activities of the corresponding kinases, CDK2 and CDK4 [44,51]. In addition, the HDAC inhibitors may increase the stability and transcriptional activities of RUNX3, which mediates induction of p21 and product of anti-apoptotic gene *Bim* (Bcl-2-interacting mediator of cell death) [52,53,54,55].

### 4.2 Apoptosis Induction

HDAC inhibitors induce apoptosis in tumor cells by regulation of pro-apoptotic and anti-apoptotic genes (for a review see [56,57,58]). The mechanisms by which different HDAC inhibitors induce apoptosis include activation of both extrinsic and intrinsic apoptotic pathways.

Initiation of the extrinsic apoptotic pathway by HDAC inhibitors was proven in many in vitro experiments. HDAC inhibitors have been demonstrated to influence death receptors TRAIL (TNF related apoptosis inducing ligand), DR5 (Death receptor 5), Fas (TNF superfamily 6), TNF (Tumor necrosis factor) and TNF-related ligands Fas-L, LIGHT (TNF superfamily member 14) and TLA1 (Transparent leaf area peptide). Inhibition of those death receptors and their ligands inhibits apoptosis induced by HDAC inhibitors [57,59,60,61]. In vivo experiments with xenograft using tumor cells with TRAIL and Fas suppressed by siRNA showed a significant decrease in apoptosis after treatment with VPA [62]. HDAC inhibitors also activate intrinsic apoptotic pathway. They regulate transcription of pro-apoptotic genes such as *Bid* (BH3 interacting domain death agonist protein), *Bad* (Bcl-2 associated agonist of cell death protein) and *Bim* that activate the intrinsic apoptotic pathway [42,58,63,64].

It can be concluded that in tumor cells exposed to HDACs inhibitors pro-apoptotic genes involved in the extrinsic (*TRAIL*, *DR5*, *FAS*, *FAS-L*, and *TNF-α*) and/or intrinsic apoptotic pathways (*BAX*, *BAK* and *APAF1*) are up-regulated, while anti-apoptotic genes (*Bcl-2* and *XIAP* (X-linked inhibitor of apoptosis protein)) are downregulated [10]. HDAC inhibitors can, however, enhance the levels of anti-apoptotic protein Bcl-2 via activation of ERK [65]. Besides, these effects on gene expression, the HDAC inhibitors increase amounts of reactive oxygen species (ROS) that can induce apoptosis in leukemic cells (Jurkat, ML-1, U937, HL-60, K-562, CEM-CCRF and its doxorubicin selected *P*-glycoprotein overexpressing subline and FDC-P1 and sublines overexpressing Bid, Bcl-2 and Bid plus Bcl-2) [63,66,67]. SAHA and entinostat increase expression of binding protein-2 (TBP-2) that inhibits thioredoxin in LNCaP prostate cancer, T24 bladder cancer and MCF7 breast cancer cells [68]. Thioredoxin is an intracellular antioxidant, therefore treatment of tumor cells by these HDAC inhibitors induce ROS-dependent apoptosis [69,70]. We found that VPA induces apoptosis more effectively under hypoxic conditions and overcomes hypoxia-induced resistance to cisplatin (CDDP) in high risk neuroblastoma derived cells UKF-NB-3 and CDDP resistant subline [71] probably by induction of HIF-1α degradation [72].

### 4.3. The Effects on Induction of Autophagy

Acetylation of many autophagy-related proteins, such as the product of the autophagy-related genes (*ATGs*), is regulated by the balance between HATs and HDACs [73]. Autophagy can also be regulated through the acetylation of transcription factors such as FOXO [74]. Different HDACs influence autophagic activity by different mechanisms as showed the results of several experiments described below. HDAC6 induces autophagy when the ubiquitin-proteasome system (UPS) is impaired. Knockdown of HDAC2 inhibits autophagy in cardiomyocytes [75]. On the contrary, inhibition or knockdown of HDAC1 in HeLa cells promote formation of autophagic vacuoles [76]. HDAC10 induces autophagy-mediated cell survival in neuroblastoma (E(2)-C, Kelly, and IMR32 cells) and its inhibition sensitizes cells to cytostatics [77]. SIRT1 forms a complex with components of the autophagy machinery (Atg5, -7, and -8) and stimulates autophagy [78].

The role of HDAC inhibitors induced autophagy in the death of cancer cells is still controversial. Some studies have reported that autophagy serves as a cell death mechanism as either autophagy inhibitors or knockdown of ATGs reduces the anticancer effect of HDAC inhibitors. Moreover, using different in vivo models, a combination of HDAC inhibitors and autophagy inhibition reduces colon cancer cells HCT116 growth [79]. In contrast, autophagic degradation of intracellular components is a death signal and leads to the cytotoxic effect of autophagy. For example, in panel of hepatocellular carcinoma cells SAHA-induced cytotoxicity is inhibited by 3-methyladenine, which inhibits autophagy by blocking the autophagosome formation via the inhibition of class III PI3K, or by *Atg5* knockout [80]. Cell death in endometrial stromal sarcoma cells induced by SAHA is caused by autophagy [81]. SAHA induces apoptosis in *TP53* wild type cancer cells, while the absence or degradation of cytoplasmatic p53 leads to activation of the autophagic pathway which consequently induces cell death [82]. The above-mentioned discrepancies might be due to differences in the used models, cancer cells, HDAC inhibitors and their doses.

Several signaling pathways play a role in the induction of autophagy by HDAC inhibitors. mTOR (Mechanistic target of rapamycin) is one of the most important suppressors of autophagy via phosphorylation and inactivation of the ULK1 (Unc 51 like autophagy activating kinase 1) complex which is an upstream component of the autophagy pathway. mTOR inactivation by SAHA restores functions of ULK1 [80,83,84]. Overexpression of ATG genes induced by SAHA is caused by the stimulation of NF-κB activity via modulation of RelA/p65 (NF-κB p 65 subunit) signaling [85]. Some studies also show that SAHA cause autophagy by ROS production in leukemic and hepatocellular carcinoma derived cells [84,86]. The fact that some HDAC inhibitors can induce cell death in the apoptosis-resistant cells by induction of autophagy seems to be the important feature for clinical practice. Romidepsin and HDAC1 siRNA induce autophagy in HeLa cells [76]. SAHA inhibits growth of short term culture glioblastoma cells xenografts in nude mice by autophagy induction via downregulation of AKT-mTOR signaling [87]. Based on the above-mentioned facts, we can suppose that induction of autophagy by HDAC inhibitors may be a promising therapeutic anticancer strategy. 

### 4.4. The Effects on Non-Coding RNA

HDAC inhibitors have been described to alter non-coding RNA expression. TSA and SAHA induce miR-129-5p overexpression and apoptosis in thyroid cancer cells. miR-129-5p alone induces cell death and knockdown experiments showed that its expression is necessary for HDAC inhibitor-induced cell death [88]. Treatment of breast cancer cells and normal human fibroblasts with a combination of sodium butyrate and panobinostat increased the expression of miR-31 which induces cellular senescence via inhibition of BIM1 (Bisindolylmaleimide-based protein kinase C inhibitor 1) [89]. This was demonstrated by transfection of cells by vector overexpressing BMI1 and on the other hand by its knock down by BMI1 shRNA. HDAC inhibition in B-cell lymphomas cells (Daudi, Ramos, Raji, Su-DHL-6, OCI-Ly-19 and OCI-Ly-3) and non-malignant B cells silences Myc-mediated transcriptional repression of the miR-15 and let-7 miRNA families, which induces apoptosis by downregulation of the anti-apoptotic genes *Bcl-2* and *Bcl-xL* and, thus, activates apoptotic pathways [90]. HDAC1 enhances miRNA processing by acetylating protein DGCR8 (DiGeorge critical region 8 protein) which processes miRNA and this acetylation increases the production of mature miRNAs in embryonic kidney cells and acute myeloid leukemia cells (AML) [91]. Moreover, several HDACs are also targeted by miRNA, e.g., miR-449a regulates prostate cancer both androgen dependent and independent cells (PC-3, DU-145, BHP-1 and LNCaP) growth and viability by targeting and repressing HDAC1 [92].

Long non-coding RNAs (lncRNAs) are important cellular regulator molecules at the transcriptional and post-transcriptional level. They exhibit a wide spectrum of functions such as regulation of alternative splicing, transcriptional patterns, and protein activity. They also have epigenetic effects and are precursors of miRNAs [93]. One of the mechanisms of transcription regulation by lncRNAs is recruiting histone modifying complexes (e.g., PRC (Polycomb repressive complex)) to target loci which are either activated or silenced, depending on the histone marks [94].

The data of Yang et al. showed that approximately 5% of intergenic lncRNA and about 6% of protein coding genes were significantly differentially expressed in hepatocellular cancer cell lines (Huh7, Bel7402, Bel7721, and HepG2) after treatment with TSA [95]. Intergenic lncRNAs regulate gene expression through modifying the chromatin complexes or RNA binding proteins and their aberrant expression is associated with cancer [96].

Low expression of lncRNA Xist (X inactive specific transcript which plays a major role in the X inactivation process) seems to be a predictor of efficacy of abexinostat in breast cancer cells [97]. One may speculate from the above-mentioned facts that changes of lncRNAs expression may be one of the mechanisms of the anticancer effect of HDAC inhibitors. However, further studies with non-coding RNAs are necessary.

### 4.5. The Effects on Cellular Signaling Pathways

Another mechanism of anticancer effects of HDAC inhibitors is regulation of cell differentiation by activation of some of protein kinases (i.e., ERK). Protein kinases modulate biological processes like cell growth, differentiation and apoptosis. HDAC inhibitors were found to increase DNA binding and transactivation activity of AP-1 transcription factor *via* ERK activation, increase expression of c-Jun (Jun activation domain binding protein) and induce its phosphorylation in SH-SY5Y neuroblastoma cells [65,98]. Although it is not yet clear how HDAC inhibitors affect ERK, it is supposed that they can induce synthesis of a still unknown factor that activate the ERK signaling pathway [65] or is incorporated into phospholipid molecules that activates ERK via the phosphatidylinositol 3-kinase (PI-3K)/Janus kinase 2 (JAK 2)/MEK-1-dependent and the tyrosine kinase-Ras-dependent pathways [99,100]. HDAC inhibitors also increase the expression of genes that are involved in regulation of ERK/AP-1 signaling, for example the growth associated protein-43 (*GAP-43*) and *Bcl-2*, and hence they might increase growth of some cancer cells, e.g., in SH-SY5Y neuroblastoma cells or immature cortical neuroblasts in GAP-43^−/−^ mice [65,101].

VPA also affects Wnt signaling that is due to phosphorylation of serine 9 in the glycogen synthase kinase-3β (GSK-3β) [102]. The Wnt signaling pathway plays an important role in various cancers such as colon, breast, ovarian, prostate and endometrial cancers as well as medulloblastoma and melanoma [103]. Inactivation of APC (Adenomatous polyposis coli) functions and of β-catenin induces overexpression of the HDAC2 which protects colorectal cancer cells HT-29 from death [10]. Therefore, we may speculate that HDAC2 is a target of APC/β-catenin. Moreover, HDAC inhibitors decrease polyp generation in a colon carcinoma model of APC deficient mice, probably via damage of the HDAC2 by degradation in proteasomes [10]. Furthermore, VPA increases proliferation and self-renewal of normal hematopoietic stem cells by inhibition of GSK-3β that activates the Wnt pathway [104].

Acetylation of p53, which may be caused by HDAC inhibitors, decreases generation of a complex p53/Mdm2 E3 ligase, whereas hypoacetylation increases its degradation by the proteasome and cancels p53-mediated growth arrest and apoptosis as demonstrated by experiments with human non-small cell carcinoma cells H1299 transfected by different *Tp53* mutants [48]. In addition, acetylation induced by HDAC inhibitors influences the expression of several proteasomal enzymes (Ubc8 E2 ubiquitin conjugase, RLIM-subunit of the SCF E3 family of the ubiquitin ligase) in human embryonal kidney cells [105].

### 4.6. The Antiangiogenic Effect

HDAC inhibitors can affect cancer angiogenesis and inhibit cellular stress response pathways, thereby interfering with the metastatic process. The anti-angiogenic effects are associated with down-regulation of pro-angiogenic genes such as the genes of the vascular endothelial growth factor (VEGF) and/or endothelial nitric oxide synthase (eNOS) [106,107,108]. Expression and enzymatic activity of eNOS are influenced by its phosphorylation which is catalyzed by Akt and numerous hemodynamic and hormonal stimuli [109,110,111]. HDAC inhibitors decrease the stability of eNOS mRNA by binding to the 5′-untranslated region of eNOS mRNA [112]. In addition, HDAC inhibitors decrease the levels of the VEGF receptors in neuroblastoma cells [113].

Moreover, HDAC inhibitors induce hyperacetylation of HIF-1α, a pro-angiogenic transcription factor, which induce its degradation [72]. VPA decreases angiogenesis by enhancing production of the anti-angiogenic proteins thrombospondin-1 and activin A via downregulation of pro-angiogenic factors such as the basic fibroblast growth factor (bFGF) [114]. Expression array showed that in prostate cancer cells VPA induces the up-regulation of *TSP1* (*Thrombospondin-1*), multiple *TIMP* (*Tissue inhibitor of metalloproteinase*) isoforms and *TGFβ* (*Transforming growth factor β*), while expression of *IGF1* (*Insulin like growth factor 1*) and *VEGF* is decreased [115]. VPA and TSA decrease the formation of capillary tubes of HUVEC (human vascular endothelial cells), but they do not induce their apoptosis [116].

On the contrary, Lin et al. found that HDAC inhibition resulted in the development of metastatic phenotype including increasing of matrix metalloproteinases (MMPs) expression in some cancer cell lines (liver (SNU-398, Huh6, HepG2, Hep3B, Huh7, PLC5, HCC36, TONG, HA59T, Sk-hep-1, HA22T, and Malhavu), lung (H358, H1437, H661, H226Br, H1299, CL1-3, CL1-0, H23, H928, and A549), gastric (NUGC, SC-M1, AZ521, AGS, and HR), and breast *(*MDA-231, Hs578T, and MCF-7) cancer) [117]. Degradation of extracellular matrix by MMPs is important for angiogenesis, tissue invasion and metastasis.

### 4.7. HDAC Inhibitor-Induced Modulation of Immune Response

A decrease in HDAC activity alters expression of MHC (Major histocompatibility complex) and costimulatory molecules [118,119]. The subsequent increase in immunogenicity intensifies activation of T cells and the prolonged survival of experimental animals [120,121]. Moreover, inhibition of HDAC6 activates naïve T cells [119,120]. In addition, HDAC inhibitors influence different lymphocyte populations. Inhibition of Class II HDACs enhances number and function of Treg and Class I HDAC inhibitors enhances the functions of NK cells and CD8 T cells [122].

After exposure to SAHA or entinostat breast (MDA-MB-231), prostate (LNCaP) and pancreas (AsPC-1) carcinoma cells are more sensitive to T-cell-mediated lysis in vitro [123]. This increased immune reaction is directed against the HLA (Human leukocyte antigens) class I/epitope complexes and the increased sensitivity to antigen-specific cytotoxic T-lymphocyte lysis indicate that HDAC inhibition induces immunogenic modulation by promoting a signature of immune recognition. Several tumor associated antigens have been shown to be epigenetically silenced in malignancies impede immune recognition by cytotoxic T cells and contributing to worse prognosis [124,125]. The exposure of human carcinoma cells to SAHA has previously been shown to result in upregulation of HLA related genes [126].

### 4.8. The Effects on Stem Cells

Epigenetic changes are important for reprogramming of somatic cells into pluripotent stem cells. Therefore, several inhibitors of epigenetic-modifying enzymes including HDAC are able to reprogram somatic cells into the pluripotent stem cells by modifying a chromatin structure and making it more permissive to transcription factors [127]. There was described amplification and maintenance of normal human hematopoietic stem cells induced by HDAC inhibitors [128], enhancement of the epithelial–mesenchymal transition of colorectal and breast cancer cells [129,130] and induction of CD133 (a marker of cancer stem cells in some cancers including the brain tumors) expression in human glioma [131]. The experimental in vitro study showed that HDAC3 promoted self-renewal of glioma stem cells and those experimental results were proved also by the studies utilizing the tumor samples [132]. Our recent study shows that HDAC inhibitors (VPA, SAHA, and MS-275) increase CD133 significantly in neuroblastoma cell lines that do not show methylated its CpG promoters and VPA treatment may increase CD133+ cells that show higher resistance to cytostatics than CD133− cells. This increase, found in the CD133+ cells was not caused by elimination of CD133− cells. Moreover, VPA treatment enhanced clonogenicity and the ability to generate neurospheres, increased Akt phosphorylation and induced expression of the pluripotency transcription factors (Oct-4 (octamer-binding transcription factor 4), Nanog (Homeobox Transcription Factor Nanog), Sox2 (sex determining region Y)) [133]. Similar increase of CD 133+ cells was found also in glioblastoma cells treated by VPA [134]. Amplification of cancer “stem like cells” might be unwanted action of HDAC inhibitors.

### 4.9. Other Effects of HDAC Inhibitors

Several HDAC inhibitors are known to be metabolized by cytochrome P450 (CYP) and they also influence expression of the CYP enzyme proteins and their enzyme activities. Since CYP enzymes are involved in biosynthesis and metabolism of many endogenous physiologically active substances and drugs, this mechanism may be involved in potentiating of the effects of some anticancer drugs by HDAC inhibitors. We found that VPA and TSA change CYP enzyme expression in neuroblastoma cells [135].

Several proteins important for DNA repair e.g., Ku70, flap structure-specific endonuclease 1 (FEN1), Werner syndrome (WRN), ataxia teleangiectasia mutated protein (ATM), mediator of DNA damage checkpoint 1 (MDC1) and DNA-dependent protein kinases (DNA PK) are regulated by acetylation which may be increased by HDAC inhibitors [136]. HDAC1 and 2 inhibition decreases the DNA repair processes mediated by BAL-associated protein (BBAP) which protects cells against DNA-damaging agents [137]. HDAC6 and SIRT2 are able to deacetylate α-tubulin and so stabilize microtubules [138].

HDAC inhibitors have also anti-parasitic activity, e.g., against Plasmodium and Trypanosoma [139].

**Figure 1:**
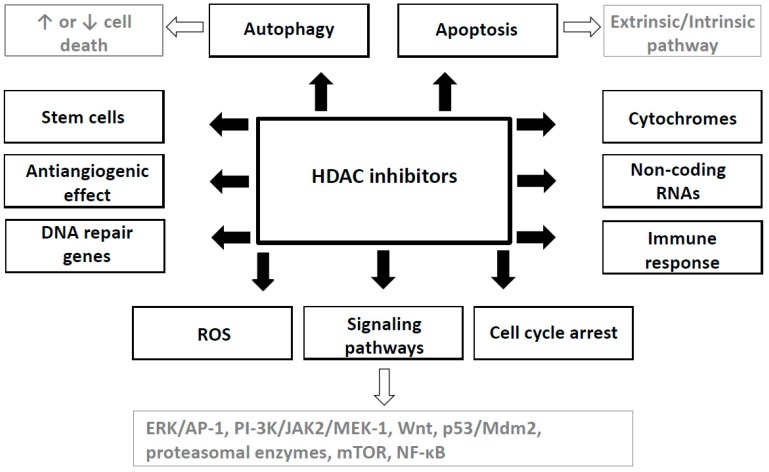
Mechanism of anticancer effects of HDAC inhibitors. ROS: reactive oxygen species.

Above mentioned mechanisms of HDAC inhibitors (see Figure 1 and Table 2) are involved in different HDAC inhibitors and in different cancers to varying degrees.

## 5. Combination of HDAC Inhibitors with Other Anticancer Therapeutic Modalities—Preclinical Studies

The results found in the in vitro and in vivo experiments using various cancer cells have demonstrated that combination of HDAC inhibitors with anticancer drugs and/or radiotherapy have synergistic or additive effects [37,140] (see Table 3). Therefore, combinations chemotherapy and/or radiotherapy with HDAC inhibitors have also been used in clinical trials [141].

HDAC inhibitors were combined with other epigenetic modifiers. Inhibitors of DNA methyl transferases azacitidine and decitabine had increased antitumor effects on myelodysplastic syndrome, prostate, ovarian and pancreatic endocrine tumors cells when used with HDAC inhibitors [52,55,142,159,160]. Decitabine and VPA both induced apoptosis and the combination increased their effects both in vitro and in vivo on leukemic cells and on medulloblastoma and rhabdomyosarcoma that develop in *Ptch1 (Protein patched homolog 1)* knockout mice [143,144]. On the contrary, this combination induced the CD133 expression in neuroblastoma cells with the methylated CD133 promotor [133]. Co-treatment of several cancer cells (prostate, pancreatic, lung and AML) with TSA and decitabine synergistically induced apoptosis [52,55,145,146]. In addition, tranylcypromine (monoamine oxidase inhibitor) and SAHA showed synergistic enhancement of apoptosis in glioblastoma cells [58].

Positive effects have been reported for combinations of HDAC inhibitors and ROS-generating agents. Adaphostin (a tyrosine kinase inhibitor that induces intracellular ROS) potentiates entinostat and SAHA induced apoptosis in leukemia cells and depletion of ROS scavenger GSH potentiates the anti-leukemic effect of SAHA [58,147]. Panobiostat sensitized lung adenocarcinoma cells including cells with *K-ras* (Kirsten rat sarcoma viral oncogene homolog) or epidermal growth factor receptor (*EGFR*) mutation to the anti-proliferative effects of the tyrosine kinase inhibitor erlotinib in the in vitro experiment [148]. EZH2 (catalytic subunit of polycomb repressive complex 2) interacts with class I HDACs and transcriptional repression by EZH2 requires the activity of the HDACs and HDAC downregulates PRC2 proteins. Therefore, one may speculate that concurrent inhibition of these epigenetic silencing enzymes has synergistic antitumor effects. Co-treatment non-small cell lung cancer (NSCLC) cells with 3-deazaneplanocin A (EZH inhibitor) and vorinostat synergistically induced apoptosis in all tested NSCLC cell lines, independently on their *EGFR* status. The co-treatment by EZH and HDAC inhibitors induced accumulation of p27Kip (Cyclin dependent kinase inhibitor p27) and a decrease in cyclin A, suppressed EGFR signaling, both in *EGFR*-wild-type and mutant NSCLC cells [149].

Another effective combination of HDAC inhibitors is that with proteasome inhibitors. Cancer cell death due to a combination of proteasome and HDAC inhibitors is caused by induction of oxidative stress, endoplasmic reticulum stress and stimulations of JNK (Jun-N-terminal kinase). Treatment of multiple myeloma cells with bortezomib made the cells more sensitive to HDAC inhibitors [150]. Trials in patients with multiple myeloma demonstrated an increase in SAHA antitumor effects in combination with bortezomib [161,162]. Proteasome inhibitor marizomib potentiates apoptosis induced by SAHA or entinostat in pancreatic cancer cells [151]. The in vitro and in vivo studies with lymphomas cells including bortezomib resistant ones showed that proteasome inhibitor carfilzomib increased the anticancer effect of SAHA [152,153].

Numerous studies show synergisms or additive effects of HDAC inhibitors and DNA damaging agents such as topoisomerase inhibitors, DNA intercalators, inhibitors of DNA synthesis, covalently modifying DNA agents (i.e., doxorubicin, epirubicin, etoposid, CDDP, melphalan, and temozolomide) and ionizing radiation in many cancer cell lines (reviewed in [37]). VPA combined with CDDP or etoposide (VP-16), but not with vincristine, was found to act synergistically. These results indicate that HDAC inhibitors increase the cytotoxic efficiency of the only of several DNA-damaging anticancer drugs. The mechanisms of the potentiating effects of HDAC inhibitors have not yet been fully explained. The sequence of drug application is important for sensitizing neuroblastoma cells to CDDP and VP-16 by VPA. It potentiates the cytotoxic effect of CDDP or VP-16 only when added simultaneously, or when cells were preincubated with cytostatics before exposure to HDAC inhibitors. In contrast, the reversed sequence (pretreatment of cells with VPA) did not give any further increase in cytotoxicity of cytostatics [154]. The results found by Luchenko et al. [155] indicated that DNA relaxation is not required for the synergy of belinostat and romidepsin with CDDP and VP-16 in small cell lung cancer cells. One can speculate that the changes in the structure of DNA caused by CDDP and VP-16 (formation of DNA adducts or DNA cross-links by CDDP, intercalation of VP-16 into DNA, and formation of ROS by both drugs) increase accessibility of nucleosomal core histones to their acetylation, which additionally determines transcription of some genes involved in DNA repair or apoptosis. Kim et al. described that pre-treatment of cells (glioblastoma lines D54 and U118, breast cancer MCF-7, normal breast MCF-12F and intestinal FHs74Int) with SAHA or TSA enhances the cytotoxicity of VP-16, ellipticine, doxorubicin, and CDDP but not that of drugs which do not target the DNA, such as 5-fluorouracil [156]. It only partially agreed with our results, Kim et al. [156] found potentiation of cytotoxicity after pretreatment of cells with HDAC inhibitors before exposure to other drugs, while the results of our experiments indicated the opposite phenomenon; the administration of VPA after DNA-damaging cytostatic increased their cytotoxicity [154]. Nevertheless, both groups used different HDAC inhibitors and different cell lines.

HDAC inhibitors are able to deacetylate α-tubulin which has stabilizing effect on microtubules. There was found that combination of TSA and paclitaxel increases apoptosis induction in endometrial and anaplastic thyroid carcinoma cells [138,157].

The in vitro experiments and the experiments with mice in vivo showed that the combination of VPA and temozolomide enhanced the apoptotic and autophagic cell death, as well as suppressed the migratory activities in temozolomide-resistant glioblastoma cells that express *O-*(6)-methylguanine-DNA methyltransferase (MGMT). The effect of VPA is caused by downregulation of MGMT [158].

## 6. Clinical Studies and Registered Drugs

As mentioned above, several HDAC inhibitors are tested in clinical studies and four of them are approved drugs (vorinostat, belinostat, panobinostat and romidepsin). The most common toxicities of those HDAC inhibitors, which are not the class-specific and have been observed in all their types, are thrombocytopenia, neutropenia, diarrhea, nausea, vomiting and fatigue [163].

SAHA had only the modest activity as a single agent (response rate is 10–20% in AML and myelodysplastic syndrome (MDS)). However, a combination with 5-azacitidine increased its response rate by 30%. The combination of SAHA with idarubicin and cytarabine had synergistic activity that was maximal when SAHA preceded cytarabine and there were responses in all patients with AML with FLT3/ITD mutation, which is otherwise associated with a worse prognosis [164,165]. Administration of SAHA in refractory CTCL patients showed in phase II trials objective response in nearly 30% [166,167]. HDAC inhibitor silences the genes in common translocations associated with hematological malignancies, e.g., those of the AML/ETO fusion protein [168]. Phase I study of patients with advanced leukemia and MDS treated with SAHA showed clinical benefit in AML and MDS [169]. The clinical phase II study proved that panobinostat is effective in relapsed/refractory Waldenstrom macroglobulinemia [170]. Mocetinostat had the only limited efficacy as showed the clinical study phase II for the treatment of patients with refractory chronic lymphocytic leukemia (CLL). Therefore, the combination with other agents such as conventional chemotherapeutic drugs was recommended [171]. Panobinostat underwent phase I and II clinical studies for the treatment of both solid and hematologic malignancies and phase III clinical trials for CTCL and chronic myeloid leukemia that showed promising results against CTCL [172] and leukemias, respectively, and proved the increased acetylation of histones in malignant cells that was associated with apoptosis [173]. Panobinostat also underwent phase III clinical trials against CTCL and leukemia in an oral form and showed the positive effect. Despite the promising results in the treatment of CTCL, SAHA and romidepsin have not been effective in studies with different solid tumors (neuroendocrine tumors, glioblastoma multiforme, mesothelioma, refractory breast, colorectal, NSCLC, prostate, head and neck, renal cell, ovarian, cervical and thyroid cancers) and Hodgkin lymphoma [174,175].

Therefore, the clinical trials tend to combine HDAC inhibitors with other drugs to enhance their anticancer effects. A clinical study showed that VPA increased efficacy of radiochemotherapy with temozolomide in glioblastoma patients [176]. VPA with doxorubicin appeared to have the 16% response rate in patients suffering from refractory or recurrent mesothelioma [177]. SAHA improved effect of carboplatin and paclitaxel in NSCLC [178] and reversed resistance to tamoxifen in estrogen receptor positive breast cancer [179]. Phase II study with a combination of erlotinib with entinostat showed prolonged progression-free survival in NSCLC patients harboring high E-cadherin levels, irrespective of the *EGFR* genotype compared to erlotinib monotherapy, while does not improve the outcome of patients with low E-cadherin [180]. Clinical trial with the combination of the immune checkpoint therapy with the HDAC inhibitor is ongoing [181].

## 7. Conclusions and Future Direction

HDACs are involved in different cellular pathways and functions; nevertheless, further studies are necessary to disclose all their functions and cellular interactions, which might result in development of more efficient therapy with HDAC inhibitors. HDAC inhibitors seem to be a promising group of anti-cancer drugs, particularly in combination with other anti-cancer drugs and/or radiotherapy. Of HDAC inhibitors, SAHA and romidepsin have been approved for CTCL, romidepsin also for PTCL, belinostat for therapy of PTCL and panobinostat for multiple myeloma. Many other HDAC inhibitors are in clinical trials for the treatment of both hematological and solid malignancies. Even though many biological effects of HDAC inhibitors have been found, explanations of these effects remain unclear.

In addition, their use in the combination with other drugs and the schedule of such drug combinations need to be investigated, in both preclinical and clinical studies. Indeed, recently, we have found that sequence of HDAC inhibitor and DNA-damaging drug is important for increase of cytotoxicity [154]. The discovery of predictive factors for evaluation of HDAC inhibitors is also necessary as demonstrated in the study with combination of erlotinib with entinostat [180]. The other most important question is whether the pan-HDAC inhibitors or the selective inhibitors will be more efficient in different types of cancers. Furthermore, assumption about the role of some HDAC inhibitors as inducers of cancer stem cells [133,134] or about the phenomenon that HDAC inhibition may enhance the epithelial–mesenchymal transition of cancer cells [129,130] needs further studies. We may conclude that while the preliminary results are promising, large multicentric clinical studies are needed to ascertain whether this treatment offers beneficial clinical outcomes with tolerable side-effect profiles.

However, the great potential for epigenetic therapies that is caused by the fact that epigenetic changes are reversible might be considered.

## Figures and Tables

**Table 1:** Overview of selected histone deacetylases (HDAC) inhibitors.

Class	HDAC Inhibitor	Target HDAC Class	Clinical Status
hydroxamic acids	Trichostatin A	pan	preclinical
	SAHA	pan	approved for cutaneous T-cell lymphoma
	Belinostat	pan	approved for peripheral T-cell lymphoma
	Panabiostat	pan	approved for multiple myeloma
	Givinostat	pan	phase II clinical trials—relapsed leukemia and multiple myeloma
	Resminostat	pan	phase I and II clinical trials—hepatocellular carcinoma
	Abexinostat	pan	phase II clinical trial—B-cell lymphoma
	Quisinostat	pan	phase I clinical trial—multiple myeloma
	Rocilinostat	II	phase I clinical trial—multiple myeloma
	Practinostat	I, II and IV	phase II clinical trial—prostate cancer
	CHR-3996	I	phase I clinical trial—advanced/metastatic solid tumors refractory to standard therapy
short chain fatty acids	Valproic acid	I, IIa	approved for epilepsia, bipolar disorders and migraine, phase II clinical trials—several studies
	Butyric acid	I, II	phase II clinical trials—several studies
	Phenylbutyric acid	I, II	phase I clinical trials—several studies
benzamides	Entinostat	I	phase II clinical trials—breast cancer, Hodgkin´s lymphoma, non-small cell lung cancer, phase III clinical trial—hormone receptor positive breast cancer
	Tacedinaline	I	phase III clinical trial—non-small cell lung cancer and pancreatic cancer
	4SC202	I	phase I clinical trial—advanced hematological malignancies
	Mocetinostat	I, IV	phase II clinical trials—Hodgkin´s lymphoma
cyclic tetrapeptides	Romidepsin	I	approved for cutaneous T-cell lymphoma
sirtuins inhibitors	Nicotinamide	all class III	phase III clinical trial—laryngeal cancer
	Sirtinol	SIRT 1 and 2	Preclinical
	Cambinol	SIRT 1 and 2	Preclinical
	EX-527	SIRT 1 and 2	cancer preclinical, phase I and II clinical trials—Huntington disease, glaucoma

**Table 2:** Main mechanism of anticancer effects of HDAC inhibitors—preclinical studies.

Mechanism	Target	Model	HDAC Inhibitor	Ref.
Cell cycle arrest	CDKN1A/p21	in vitro leukemic cells U937	SAHA	[42]
	CDKN1A/p21	in vitro bladder cancer cells T24	SAHA	[43]
	CDKN1A/p21	in vitro normal breast MCF-10A, prostate cancer PC-3, DU145, colon cancer SW620, ovarian cancer IGROV, breast cancer MCF-7, lung cancer A549	FR901228	[44]
	p53	in vitro colon cancer HCT116 + knock out DNMT1^−/−^, DNMT3B^−/−^, DNMT1^−/−^ and DNMT3B^−/−^ HCT116	TSA	[46]
	p53	in vitro lung cancer A549	depsipeptide	[47]
	p53	in vitro lung cancer H1299, osteosarcoma U2OS, human embryonal kidney HEK293	TSA, nicotinamide	[48]
	p21	in vitro colon cancer HCT116 + knock out p53^−/−^, p21^−/−^	sodium butyrate	[49]
	p21	in vitro gastric cancer TMK-1, MKN-1, MKN-7, MKN-28, MKN-74, MKN-45, KATO III, HSC-39, oral squamous carcinoma HSC-4, Ho-1-N-1, Ho-1-U-1	TSA	[50]
	RUNX3	in vitro pancreatic endocrine tumors- insulinoma CM, carcinoid BON, somatostatinoma QCP-1	TSA	[52]
	RUNX3	in vitro prostate cancer DU-145, LNCaP, PC-3	TSA	[55]
Apoptosis	death receptor	in vitro leukemic cells HL60	apicidin	[60]
	death receptor	in vitro AML cells from patients, normal CD34+ progenitors	MS275	[61]
	intrinsic pathway	in vitro leukemic cells U937	SAHA	[42]
	intrinsic pathway	in vitro T cell leukemia CEM-CCRF and doxorubicin derived P-gp+(CEM-P-gp)	SAHA	[63]
	intrinsic pathway	in vitro fetal lung fibroblasts IMR90, osteosarcoma U2OS and Saos-2, colon cancer HCT116 p53^−/−^	SAHA, TSA	[64]
	intrinsic pathway	in vitro leukemic cells Jurkat, ML-1	MS275	[66]
	intrinsic pathway	in vitro leukemic cells Jurkat, U937, HL-60, K562, U937 expressing Bcl-2, Bcl-X_L_, CrmA, C8DN, p21 antisense	MS275	[67]
	intrinsic pathway	in vitro prostate cancer LNCaP, bladder cancer T24, breast cancer MCF7	SAHA	[68]
	intrinsic pathway	in vitro neuroblastoma UKF-NB-4, normoxic and hypoxic condition	VPA	[71]
Autophagy	FOXO3	in vitro 293T, mice embryonal fibroblasts wild t. and SIRT^−/−^, Rat1 fibroblasts inducible expressing FOXO3	Nicotinamide, BML-210, splitomicin, TSA	[74]
	Atg5 & Beclin1	in vitro rat cardiomyocytes, in vivo model- a-MHC Beclin transgenic mice	TSA	[75]
	unknown	in vitro cervical cancer HeLa	siRNA HDAC1, FK228, SAHA	[76]
	Atg4D	in vitro neuroblastoma BE(2)-C, Kelly, IMR32	siRNA HDAC10	[77]
	Atg5, 7 & 8	in vitro cervical cancer HeLa, colon cancer HCT116- SIRT1 transfected, starvation	-	[78]
	FOXO1	in vitro colon cancer HCT116 Tp53^−/−^ and ^+/+^, mouse embryonic fibroblasts, liver cancer HepG2	TSA	[79]
	Akt/mTOR, ULK1	in vitro liver cancer Hep3B, HepG2, Huh7, mouse embryonic fibroblasts	SAHA, TSA, MS275	[80]
	mTOR	in vitro endometrial stromal sarcoma ESS-1, endometrial stromal cells HESCs	SAHA	[81]
	p53	in vitro uterine sarcoma MES-SA, ESS-1, cervical cancer HeLa, PANC-1, leukemic cells, pancreatic cancer Jurkat, HL-60, U937	SAHA	[82]
	ULK1	in vitro glioblastoma T98G, MEF cells, ULK1/2 knockout MEF, ATG3 knockout MEFs	SAHA	[83]
	ULK1	in vitro leukemic cells Jurkat	SAHA	[84]
	NF- κB	in vitro PC3, DU145, and HCT116, mouse embryonic fibroblasts and MEF Atg5^−/−^	SAHA, MS275	[85]
	Akt/mTOR	short term culture glioblastoma cells xenografts in nude mice	SAHA	[87]
Effect on non-coding RNA	miR-129-5p → GALNT1 & SOX4	in vitro thyroid carcinoma BCPAP, TPC-1, 8505C, CAL62	TSA, SAHA	[88]
	miR-31→BMI1	in vitro breast cancer MDA-MB-231, MCF7, 293T, embryonal lung fibroblasts	sodium butyrate, panobiostat	[89]
	miR-15 & let-7 → Myc	in vitro and mice xenografts leukemic cells/lymphoma Daudi, Ramos, Raji, Su-DHL-6, NIH3T3, P493-6	RGFP966, depsipetide	[90]
	Drosha/DGCR8	in vitro 293FT embryonal kidney cells HDAC1 transfected	/	[91]
	miR-449a → HDAC1	in vitro prostate cancer PC-3	siRNA HDAC1	[92]
	uc002mbe.2 lncRNA	in vitro liver cancer Huh7, Bel7402, Bel7721, and HepG2, ShRNA uc002mbe.2 lncRNA	TSA	[95]
	lncRNA Xist	in vitro stem cells from 16 breast cancer cell lines	SAHA, VPA, abexinostat	[97]
Effect on signal pathways	c-Jun	in vitro neuroblastoma SH-SY5Y	VPA	[98]
	ERK	in vitro neuroblastoma SH-SY5Y	VPA	[65]
	APC/β-catenin/c-Myc	in vitro colon cancer HT-29 HDAC2 transfected, mice C57BL/6J-*APC*-/-	VPA	[10]
	GSK3 β	in vitro hematopoetic stem cells	VPA	[104]
	ubiquitin–proteasome	in vitro embryonal kidney cells HEK293T	VPA, TSA	[105]
Anti-angiogenic effect	Gja1, Irf1, Gbp2	in vitro embryonic stem cells HDAC1 mutated and wild type	/	[106]
	ACNA2D2	HDAC1 or HDAC2 mutated C57BL/6 mice	/	[107]
	PPAR, ERR	HDAC3 conditional C57BL/6 mice	/	[108]
	eNOS, Akt	in vitro endothelial cells, Akt transfected, mutatnteNOS	/	[110]
	eNOS	in vitro HUVEC	TSA, MS275	[112]
	MMP-2/VEGF	in vitro melanoma A375, toxicity tests mice	Compound 8	[119]
	semaphoring III	in vitro HUVEC	TSA, SAHA	[113]
	thrombospondin-1, activin	in vitro, neuroblastoma BE(2)-C	VPA	[114]
Modulation of immune response	TAP1 & 2, LMP-2, tapasin	in vitro+mice xenografts mouse lung TC-1, rat pancreas D11, mouse fibroblasts A9, endothelial cells PA, mouse colinic cells LMD, mouse melanoma B16F10, B16F10/TAP-1	TSA	[118]
	MHC class I	mice C57BL and B6.CB17-Prkdc, human melanoma samples, mouse melanoma B16-F10-luc	MGCD0103, LBH589, TSA	[120]
	STAT3/IL-10	BALB/c and C57BL/6 mice, TCR transgenic mice, HDAC6 recombinant mutants	/	[121]
	Tumor associated antigens	in vitro prostate cancer LNCaP, breast cancer MDA-MB-231	SAHA, entinostat	[123]
	*MHC* genes	in vitro prostate cancer PCa and DU-145	VPA	[126]

**Table 3:** Combination of HDAC inhibitors with other anticancer therapy.

HDAC Inhibitor	Other Anticancer Therapy	Model	Effect	Ref.
SAHA, tubacin	etoposide, doxorubicin	in vitro prostate cancer LNCaP, breast cancer MCF-7, normal human foreskin fibroblast cells	Potentiation in ca cells not in fibroblasts	[140]
TSA	5-aza-2′-deoxycytidine	in vitro pancreatic endocrine cancer cells	Potentiation	[52]
TSA	5-aza-2′-deoxycytidine	in vitro LNCaP, DU-145, and PC-3 prostate cancer	Potentiation	[55]
SAHA, TSA	5-aza-2′-deoxycytidine	in vitro ovarian cancer Hey, SKOv3	Synergism	[142]
VPA	5-aza-2′-deoxycytidine	in vitro leukemic cells HL-60, MOLT4	Synergism	[143]
VPA	5-aza-2′-deoxycytidine	Ptch knockout mice	Potentiation	[144]
Depsipeptide	5-aza-2′-deoxycytidine	in vitro Kasumi-1 cells and blasts from patient with t(8;21) AML	Potentiation	[145]
TSA	5-aza-2′-deoxycytidine	in vitro lung cancer A549, H719	Synergism	[146]
SAHA	β-phenylethyl isothiocyanate	in vitro leukemia cells HL60, HL60/LR, HL60/C6F, U937, ML1, samples from patients with acute myeloid leukemia	Potentiation	[147]
Panobinostat	erlotinib	in vitro lung cancer HCC827, A549, NCI-H460 (EGFR wild type and mutant)	Synergism/Potentiation	[148]
SAHA	3-deazaneplanocin A (EZH2 inhibitor)	in vitro and BALB/cAJcl-nu/nu mice xenografts lung cancer NCI-H1299, NCI-H1975, A549, PC-3	Synergism	[149]
sodium butyrate, SAHA	bortezomib	in vitro multiple myeloma U266, RPMI8226 and patient samples	Synergism	[150]
SAHA	bortezomib	in vitro and nude mice xenografts, pancreatic cancer L3.6pl, pancreatic epitelium HPDE6-E6E7	Potentiation	[151]
SAHA	carfilzomib	in vitro and nude mice xenografts, lymphoma cell SUDHL16, SUDHL4 , SUDHL6, OCI-LY10, OCI-LY3 bortezomib-resistant SUDHL16-10BR, OCI-LY10-40BR and lymphoma cells frompatients	Potentiation	[152]
SNDX-275, SAHA	carfilzomib, bortezomib	in vitro and nude mice xenografts, lymphoma cell Granta 519, Rec-1, HF-4B, JVM-2, MINO, JVM-13	Potentiation	[153]
VPA	cisplatin, etoposide	in vitro neuroblastoma cell UKF-NB-4	Potentiation-schedule dependent	[154]
belinostat, romidepsin	cisplatin, etoposide	in vitro lung cancer H82, H146, H526 and H446	Synergism-schedule dependent	[155]
TSA, SAHA	VP-16, ellipticine, 5fluorouracil, doxorubicin, cisplatin, cyclophosphamide, campthotecin	in vitro glioblastoma U118, brest cancer MCF-7, normal breast MCF-12F, normal intestinal epithelia FHs74Int	Potentiation-schedule dependent, except of 5fluorouracil, cyclophosphamide, campthotecin	[156]
VPA	paclitaxel	in vitro anaplastic thyroid carcinoma CAL-62, ARO	Potentiation	[157]
VPA	Gene therapy (MSC + HSV-TK)	glioblastoma U87 xenografts in nude mice	Potentiation	[158]

MSC + HSV-TK- herpes simplex thymidine kinase transfected bone marrow mesenchymal stem cells.
